# “The Entrepreneurial Gamer”: Regendering the Order of Play

**DOI:** 10.1177/1555412018755913

**Published:** 2018-03-12

**Authors:** Jennifer Jenson, Suzanne de Castell

**Affiliations:** 1 York University, Toronto, Ontario, Canada; 2 University of Ontario Institute of Technology, Oshawa, Ontario, Canada

**Keywords:** video game players, ludic economies, affective and immaterial labor, video games, gender

## Abstract

The designation “gamer” is structurally bound to networked economies of digital play that are rewarded fiscally, socially, and publically, an order of play that is proving difficult to overturn. That girls and women have enjoyed at best marginal positions within video game cultures is by now well recognized, yet at the very same time is too easily dismissed in light of persuasively documented increases in the numbers of women who play. This article traces a large-scale transformation of ludic engagement from participation to spectatorship that parallels the professionalizing and commodifying of traditionally embodied sports, games, and play to demonstrate how new and emerging economies of gameplay, far from opening up the playing field, threaten a further entrenchment of gendered relations.

The video game industry is a global, highly profitable market worth, according to estimates, close to US$100 billion in 2016 (Minotti, 2016). It is also an industry that, like other technology industries, employs more men than women (International Game Developers Association, 2015; Pompilio, 2016) and has had ongoing documented reports of misogyny, racism, sexism, and sexual violence against women (Cross, 2016; Jenson & de Castell, 2015; Kelleher, 2015; van der Linde, 2015). At the same time, video game culture—communities who play video games, write about them, blog about them, stream them, tweet about them, and so on—remains persistently male dominated, hostile to women and others, and deeply misogynist. While the claim has been made for at least the last 5 years by the Entertainment Software Association (ESA) that there are nearly as many females willing to self-identify as game players as male players (in 2015 that number was 41%, but it has been as high as 48%), Yee (2017) at Quantic Foundry surveyed over 250,000 players and found deep divisions in the types of games played. Women were much more likely to report that they played “family/farm simulation games” and much less likely to play “tactical shooters and sports games.” Yee’s work helps to uncover the ways in which the ESA statistics conceal a deep divide in the types of games people who identify as “male” or “female” play. Revealing this division between male and female players reinforces that exclusionary practices can and do have an impact on who plays and what they play (Gray, 2014).^1^ For example, Gray (2013) writes about how Black female players are “profiled” and harassed by male players when they speak in voice chat and that they were collectively the only group she interviewed for her research that “attempted to respond to inequality in an organized, collective manner (regardless of the outcome)” (n.p.).

Research on gender, race, sexuality, and gameplay has amply demonstrated the deeply sexist practices in the culture and industry surrounding video games (cf. Gray, 2014; Harvey & Fisher, 2015; Jenson & de Castell, 2015; Shaw, 2012, and many others). Massanari (2015) characterizes online video games, and broader technocultures such as Twitter, Reddit and 4chan, as toxic cultures that support the harassment of others. Massanari further elaborates that toxic technocultures rely on “an othering of those perceived as outside the culture…and a valorization of masculinity masquerading as a particular form of ‘rationality’” (p. 5). In other words, hegemonic masculinity is reinforced and indeed valorized through these technocultural networks to “ideologically legitimate the global subordination of women to men” (Connell & Messerschmidt, 2005, p. 832).

What is less well understood is how these inequities are, as Gill (2014) has argued “neither accidental nor incidental but produced by the labouring conditions themselves” (p. 6). This article explores the “laboring conditions” of new economies that have emerged in relation to video games. More than two decades of research on gender and technology and gender and digital gameplay give us good reason to be deeply concerned with the ways in which women (and others) have been and remain marginalized culturally, socially, and economically (Jenson & de Castell, 2011, 2013, 2015; Jenson, Fisher, & de Castell, 2011). That marginalization looks likely to worsen under conditions of professionalization, commercialization, and entrepreneurialism in and around gameplay.

## Turning to Feminism

The analysis that follows turns attention from critiques of representation to examinations of the material and economic processes that shape and constrain games, play, and players. As Hall (1997) insisted:

Representation can only be properly analysed in relation to the actual concrete forms which meaning assumes, in the concrete practices of the signifying, “reading” and interpretation; and these require analysis of the material forms…in which symbolic meaning is circulated. (p. 9)

We try to trace here the ways in which that focus by feminist and other scholars (ourselves included) on gender representation in video games has often neglected to study it as first and foremost an expression of larger and more powerful *economic* inequities. Feminist media scholarship has examined gender inequities in digital and social media consumption, use and commodification (Shade, 2014; Shepherd, 2014), as well as immaterial, affective, and aspirational labor (Banks & Deuze, 2009; Duffy, 2015, 2016; Dyer-Witheford & de Peuter, 2009; Harvey & Fisher, 2015; McRobbie, 2010; Terranova, 2000), but less attention has focused specifically on how those economies and that labor fix gender roles and expectations for who makes and plays video games—and for whom these activities accrue material rewards.

To advance beyond critical analyses of gender representation, it is helpful to interrogate the ways traditional gender inequalities are being (re)created in and through an emerging spectatorial economy of games and play. In a relatively short period of time, more and more people have tuned in to watch other people play. This is made most clear in the financially lucrative eSports contracts being awarded to mostly top male players in video game leagues (T. L. Taylor, 2012) or the top earners from video game streaming websites like Twitch and YouTube, also primarily male (Consalvo, 2017).

To better understand how material conditions for women, in particular, change when play becomes professionalized public performance, we need first to unpack what’s meant by “labor” in the immaterial, affective, and aspirational forms that characterize video game play. Then, we review the moniker “gamer” and the ways it has been used to galvanize a culture around gameplay which, even while celebrating democratic expansions of “participation,” actually retrenches gender-stratified economic privilege. Next, we examine cultures and economies that support professional play—through eSport tournaments and Twitch and YouTube broadcasters and subscribers—to show how gender inequalities are very much a part of the commodification of the ludic.

## Gendered Subjectivities: Unpacking Immaterial, Affective, and Aspirational Labor

It is beyond the scope of one short paper to detail the intricacies of labor’s transformations in the turn to the digital. It is necessary though, and helpful, to set out some terms relevant to a discussion of gender, labor, and video game cultures and industries. And while it might seem that, in a discussion of “digital work,” we overemphasize domestic labor, given this is neither a study of vacuuming or child-rearing, we do so to draw attention to continuities and convergences between those domestic practices and new forms of immaterial labor, both of which remain largely “women’s work.”

Women’s work is the unpaid social, domestic, reproductive work that is distinguished from productive, commodified labor and was consigned for that reason to relative insignificance by Marx. More recently, the value of those forms of immaterial labor whose products are not commodities has been reconceived and reclaimed as a potent form of autonomous work to contest capitalism’s economic and ideological hegemony (Lazzarato, 1996). In view of the strong participation of women and girls in media production and consumption, this reclamation of immaterial labor might give us cause to hope for more equitable terms for “women in games.”

It is along this trajectory that “participatory culture,” voluntary labor as a “gift economy,” the promotion of self-branding, and entrepreneurialism, and the valorization of “doing what you love” can be located. According to Jenkins (2006), participatory culture allows for media production on a level never before possible. Jenkins views that production as participatory; others have characterized this relation as one in which the “volunteer” or “immaterial” labors of modders, fans, volunteers, unpaid reviewers, and so on are of economic benefit to media companies (Banks & Humphreys, 2008; Postigo, 2003). Throughout these protean reconfigurations of immaterial labor, as labor practices have shifted from industrial to digital paradigms, much immaterial labor *has continued to consist* of the kind of gendered domestic work that is “used up” even as it is carried out: the cooking of food, the caring for family members, the cleaning of the house, and the kind of work that has use value, but not exchange value. Both these reproductive and affective contributions by women “on the home front” support all other economic activities, and more fundamentally still, provide the workforce with its laborers, its human resources.

The contributions of bloggers, streamers, reviewers, YouTubers, network administrators, guild leaders, modders, and the whole gamut of mostly unpaid digital media content creators, including open source programmers, have directed renewed attention to these emergent forms of immaterial labor, but in ways which fail to engage with the enduring, very substantively material reality of women’s reproductive and domestic labor, whose immateriality is of a quite different order. We contend that what else drops out in the “movement out of the kitchen and into the Internet” is recognition of the complex interplay between traditional and informatized conceptions of immaterial labor, an interplay in which gender remains a driving force of inequality, and one that goes unacknowledged so long as the focus is placed on changed forms and functions of digital labor, with women’s work (Jarrett, 2014) being “dematerialized” (Fortunati, 2007, p. 139) within a larger category which serves, in fact, to erase it. Jarrett (2014) puts the point succinctly: “It often seems as if immaterial labour was only ‘invented’ when it moved out of the kitchen and onto the Internet” (p. 15).

“Affective labor,” most simply put, is immaterial labor that produces “affects” whose distinctive contribution is the formation and maintenance of “collective subjectivities…socialities, and ultimately…society itself” (Hardt, 1999, p.89). These immaterial products of affective labor function in the constitution of alliances, affiliations, dispositions, and shared values that cohere in families, communities, political parties and, most recently, reputations, networks, and brands. “The processes of economic post-modernization,” writes Hardt (1999), “have positioned affective labour in a role that is not only directly productive of capital but at the very pinnacle of the hierarchy of labouring forms” (p. 90).

Mobilizing and then inverting the Foucauldian notion of biopower to consider “the potential of labour as biopower, a biopower from below” (p. 100), Hardt argues an *ontological* role for affective labor in contemporary capitalist society. “Biopolitical production,” he contends, “consists primarily in the labour involved in the creation of life—not in the activities of procreation but the creation of life precisely in the production and reproduction of affects” (p. 99). But from recognizing that the affective labor which can be only be accomplished through “human contact and proximity” can be accomplished through *virtual* contact and proximity as well as though material coembodiment, to arguing for “the creation of life precisely in the production and reproduction of affects” is a very big, and quite illogical, leap. So that even as Hardt clearly recognizes that “caring labour [and even more so, reproductive labour], is entirely immersed in the bodily, in the somatic” (p. 96), his argument ends by subsuming all forms of women’s work, including reproductive labor, within a category of affective labor which can be *either* actual or virtual. This is a category mistake. Affective labor may certainly create a “form of life,” but in its virtual forms, it does *not* create or sustain life—somebody still pushes the vacuum cleaner, somebody feeds the baby.

Placing primacy on digitally remediated forms of affective labor thus subordinates these gendered forms of work, which remain far away indeed from having “autonomous’ or subversive potential. As McRobbie (2010) and others have argued, the embracing of immaterialism, even as it far better comprehends the character, functions and uses of digital labor, has again diverted attention from immaterial labor’s persistently gendered divisions.

The professionalization of digital gameplay and the democratization of media content creation are immaterial, to be sure, but in fundamentally different ways from gendered immaterial and affective labor, which remain largely untouched by the purported transformations of the digital. That’s because digital content creation, which can have both use value (the pleasure of doing what one loves) and exchange value (income generation, reputational rewards, and not insignificantly, labor force support in the form of information, knowledge, and skills provision), does not, of course, contribute to the creation and support of the labor force. Those reproductive contributions of women’s work even today remain very much embodied and have in fact not been greatly assisted by digital technologies—the vacuuming still needs doing.

Contemporary efforts to formulate a conception of immaterial labor that illuminates the perils and the promise of emerging forms of the (mostly) voluntary work of creative and cultural digital production still continue to elide the persistently gendered division of immaterial labor very much materially rendered, which remains prerequisite to digital content creation in the fundamental sense that its creators are embodied human beings whose very survival depends upon the uninterrupted provision of domestic labor—and that is still work done primarily by women, and without pay.

The concept of affective labor exhibits a similarly slippery ambiguity, insofar as it fails to recognize that much affective work “in the bodily mode” cannot be reduced to or replaced by affective work accomplished virtually. That conceptual conflation in turn deflects attention from the ways digital media within a postindustrial information economy are produced collectively but selectively compensated along clearly gendered lines.

Those newly professionalized forms of ludic labor have been inelegantly designated as “playbour” (Küchlich, 2005; N. Taylor, Bergstrom, Jenson, & de Castell, 2015) and “prosumption” (Humphreys & Grayson, 2008; Ritzer & Jurgenson, 2010). A more useful refinement of our conceptual tool kit for seeing “under the hood” of immaterial work for which one hopes to be “paid to play” is provided by Duffy (2016), whose analysis of aspirational labor explicitly identifies gendered inequalities in resources, risk, time, and compensation.

Drawing on in depth interviews with 18 young “fashion bloggers,” Duffy (2016) examined the ways young fashion bloggers make sense of, articulate, and find value in their “participatory” activities of media production. She characterizes the specific form of immaterial labor in which they (and other digital content producers) participate as “aspirational labor” and further characterizes aspirational labor as a “highly gendered, forward-looking entrepreneurial enactment of creativity” (p. 3). However, discourses on aspirational labor, Duffy (2016) observes, in their invocations of passion, authenticity, community, and doing what you love not only render that labor invisible but also obscure the kinds of capital (both social and economic) needed to pursue the technology-intensive, effortful, and costly activities of doing what you love in the hopes of the kind of success that will pay you for it. “Aspirational laborers,” she goes on to explain, “pursue creative activities that hold promise of social and economic capital; yet the reward system for these aspirants is highly uneven” (p. 453). This article is asking similar questions—whose aspirational labor is rewarded and in what forms both social and economic?

Mobilizing Duffy’s arguments to address video game play in particular helps make sense of how and why increasing the numbers of women who participate in remunerative play and content creation might not necessarily mean that game culture is becoming more inclusive or equitable. Just as game journalists and scholars encounter very different rewards and punishments for doing or writing the very same things, so game playbourers and prosumers occupy very different worlds even when they inhabit the very same times and places. The next section turns to identity and the identificatory politics that surround who is recognized as someone who plays video games.

## Toxicity Online and Off: Video Game Culture Versus “Women”

Both players and makers of video games have been embroiled publically in a battle since the fall of 2014 over the roles of women and others in video game’s culture and industries. The battleground? Online—Twitter, 4chan, reddit, and 8chan—where countless individuals mounted attacks against women who dare to make and critique video games. This public “war,” which at the time of writing in 2017 has somewhat lost its momentum, is referred to widely as Gamergate. Its histories and stories have been well rehearsed by academics and the mainstream press (Braithwaite, 2016; Chess & Shaw, 2015; Cross, 2016; Gray, 2016), covering the victims of cyberattacks, online bullying and harassment, and real-world everyday harm directed at, principally, people who identify as women (cf. Collins, 2014; Edidin, 2014; Farokhmanesh, 2014). With Gamergate, the seamy underbelly of video game culture was finally revealed for what it was: misogynist, sexist, vitriolic, and hate filled. Gamergate also helped to shine a light on a video game industry in which women are, as in other technology industries, significantly underrepresented, and in steadily diminishing numbers as the job grade rises, and where they report that they deal with harassment (sexual and other) daily.

Preceding Gamergate was a hashtag that trended on twitter for many months in 2012, #1reasonwhy (there aren’t more women in the games industry), where women tweeted the harassment that they had experienced from male colleagues and friends working in the industry. That led to a panel “#1reasonwhy” at the major industry “Game Developers Conference” (GDC) in San Francisco in which leading female figures in the industry discussed their own past experiences and proposed solutions to the problem. The point here is that there is a long history in video game cultures and industries regarding explicit sexual harassment and misogyny directed at women who work, play, and write about video games—nothing new here, in fact. Except that the groundswell of fury, one way or another, over “Gamergate,” a discourse of symptoms that pitches “gamers” against women, focuses critical attention on superficialities of identificatory politics, and has in that way served as a powerful distraction from interrogating more deeply the economic forces that structure and bind digital play, its players, and its industries. That is not to say that important cultural and social issues haven’t been raised in response to Gamergate, but more that it *also* served to obscure who is remunerated and who benefits socially and economically from game making.

### Gamer Girls: Geek Fantasy and “Real Players”

This is expressed in discourses around gamers, gamer girls/z, girls who play games, and games for girls. “Gamer girls” are shorthand for girls and women who play games. It also has darker connotations beyond its simple referent—a gamer girl can be someone who just plays because her boyfriend plays, she is likely less skilled than male players, or “just holding the controller” for her “boyfriend,” and she is a loosely parodied, male gamer geek, fetish object—imagine a female that is both beautiful and skilled at playing video games. That last connotation is reinforced on YouTube and Twitter, where (mostly) young women upload images/videos that sometimes call into question that heteromale fantasy or, more often, simply reinforce it through posed images targeted to a male gaze. The Twitter hashtag #gamergirls is a good example of the messiness of the term—it has booty shots, carefully framed photos of attractive women playing, parodic calls to what a “real girl gamer is,” as well as invitations to play by women who are seeking other women to play with. There are many other examples of the use of gamer girls to demarcate women and girls who play video games—a subreddit is dedicated to the topic (http://www.reddit.comr/GirlGamers), a Steam community publically supporting female gamers that has chat and forum rules to protect its members (https://steamcommunity.com/groups/GirlGamers), and then of course there are countless YouTube videos dedicated to the topic of “fake gamer girls”—who only want a date and can’t really play, and so on.

Gamer girl is a discursive construct that signals, at its most literal, that girls and by extension women are also gamers. However, much that label might seem to interrupt the perception that gamers are white, heterosexual men, the false gender binary gamer girl evokes sets girls/women and their gameplay apart from male gamers. That distinction appeared significant especially during the height of Gamergate when Alexander (2014), a game journalist who has written for *Gamasutra* (an online publication dedicated to games and game making), *Time*, *Polygon*, and *The Guardian*, among other prominent journalistic venues, wrote a piece entitled: “‘Gamers’ don’t have to be your audience. ‘Gamers’ are over.” In it, she argues that:

‘Gamer’ isn’t just a dated demographic label that most people increasingly prefer not to use. Gamers are over. That’s why they’re so mad. These obtuse shitslingers, these wailing hyper-consumers, these childish internet-abusers—they are not my audience. They don’t have to be yours. (n.p.)

Alexander’s (2014) piece struck a chord, one that still resonates. Her piece was important because, in part, it highlighted the fact that there has been economic contraction year over year in the AAA game industry, “with only a few sterling brands enjoying predictable success.” According to Newzoo’s 2016 Global Games Market report, for the first time, mobile gaming revenues will exceed those of PC games, taking 37% of the global market share of games (https://newzoo.com/insights/articles/esports-revenues-2016-adjusted-upward-493m/). So here again we can see that in actual material terms, the market for digital games is diversifying, even as its core, white male player base is resisting. And that matters because underlying gamer girl as a discursive construct are material, social and economic inequities and inequalities that sequester gamer girls from the material and social rewards afforded to the malestream gamer.

In his 2011 book *How to do Things with Videogames*, Ian Bogost advances a similar argument to Alexander’s:

we must face a humbling perhaps even disturbing conclusion about the media forms we love: they’re just not that special…as games broaden in appeal, being a “gamer” will actually become less common if being a gamer means consuming games as one’s primary media diet or identifying with videogames as a primary part of one’s identity…. Soon gamers will be the anomaly. If we’re very fortunate, they’ll disappear altogether. Instead we’ll just find people, ordinary people of all sorts. And sometimes those people will play videogames. And it won’t be a big deal, at all. (pp. 153–154)

Wishes and wistfulness aside, both Bogost (who *hasn’t* been publically threatened and harassed for his call for the end of gamers) and Alexander argue that the medium of the video game is fundamentally changed—that players of all types, ages, genders, sexualities, and ethnicities are exploring the medium of games, not just video games but online, mobile, educational, and alternative reality games (as the success of *Pokemon Go* clearly demonstrated). Ford (2016) drawing on Roland Barthes’ piece on the death of the author called for a discursive move away from using gamer to refer to people who play video games as players:

So let’s do away with gamers. Let’s free the medium from our narrow expectations, and open our minds to the vast possibilities games offer us when we domesticate their underlying mechanisms…. The real birth of the player must be at the cost of the death of the Gamer. (n.p.)

These calls to move away from gamer constitute a deliberate *linguistic* intervention in video game culture. But this lexical shift matters only if we are also looking beyond discourse to shifts in the industry to see how gamer retains a powerful grip on game culture in its ties to burgeoning and financially lucrative industries—eSports and video game play streaming (on Twitch, Azubu, MLG.tv, and YouTube).

The common discourse for referring to eSports professionals is not in contention though—and “professional gamers” refers, of course, first and foremost to male players. But what if those were just “professional eSports players”—that really could be anyone regardless of age, sex, gender, sexual orientation, or ethnicity. Referring to “players,” can mean anyone—someone who plays board games, or someone who is a level 600 *Candy Crush* player who doesn’t pay to play, or even an 80-year-old grandmother who streams her play on YouTube.

## Reverting to Gamers: A Male-Dominated Global eSports Industry

eSports revenue is skyrocketing. Since 2014, Newzoo, a global games market research publisher, reports that it has grown from a US$194 million dollar industry to, in 2016, a US$493 million dollar industry (https://newzoo.com/insights/markets/esports/).^2^ That total includes media rights, merchandise, tickets, advertising, sponsorship, and game publisher fees. eSports has an extremely large audience, online, and in person. In December 2016, Riot, the company behind the top played and top grossing game of 2016, *League of Legends*, reported that their eSports Championship Finals were viewed by 43 million people with 14.7 current viewers during the final match (Howell, 2016). This is a major, global business, and it is growing in and through new and different business models. For instance, this year Riot, makers of the game with the largest player base worldwide, *League of Legends*, allowed fans to contribute in game items to increase the prize pool for the *League of Legends* tournament and that in-game purchasing meant that US$6.9 million was awarded in prize money, compared to the US$2.1 million the year before.

Of course many more leagues and tournaments are now part of a global eSports circuit—Major League Gaming (MLG), Electronic Sports League, Dreamhack, European Gaming League, as well as game-specific world championship leagues (*Dota 2*, *Heroes of the Storm*, *Call of Duty*, *League of Legends*, *Overwatch*), among many others that are local and extra local in the United States, Canada, China, Mexico, Europe, and Korea. This global network of eSports leagues and their increasingly corporate sponsors, including game companies, is also now supported through “mainstream” sports coverage. Both ESPN and Yahoo launched in 2016 online sections—“verticals”—devoted to eSports (Peckham, 2016; Poladian, 2015) and Activision Blizzard Inc. bought MLG for US$46 million (Gaudiosi, 2016), hoping to secure its base as an eSports global brand. What these investments point to are the growing market for eSports as an industry, and the powerful ties it is building with mainstream media (Casteillo, 2016). As the managing director for Mindshare Sportlight, a part of WPP (a marketing and communications firm), Joshua Spiegelman put it in an interview to CNBC: “The bottom line is that the core eSport consumer is not the dude playing in his mom’s basement…. A significant percentage of fans are working professionals with buying power” (Casteillo, 2016). So, who are these “working professionals” and who is their buying power benefiting first and foremost economically? As many have point out (T. L. Taylor, 2015; N. Taylor, 2016; Witkowski, 2012), they are mostly male game(r) devotees, and their buying power streams economic benefits to game companies and those who run the leagues.

eSports marks a major cultural shift from the amateur gamer to professional “player” and from play that has mostly use value (at home, with friends or without, online or off) to play as labor that has exchange value. Dyer-Witheford and de Peuter (2009) argue that the video game industry has relied heavily on immaterial labor, which is “less about the production of things and more about the production of subjectivity” (p. 4). In the case of eSports, that subjectivity is directed at the production of the “pro-gamer”—someone who is especially technically skilled at gameplay and whose play produces economic (and/or affective) value. And complementarily, it is directed at the mass production of gamers’ spectators. Küchlich (2005) characterized as playbour the work of fans who engage in play as production that is profitable for video game companies and other entertainment industries but is not necessarily financially rewarded. As Dibbell (2006) puts it, “production is melting into play” (p. 25) or as Wark (2013) offers: “you do something for nothing because you want to do it, not as labour grudgingly offered in exchange for wages or other incentives but for fun, as ‘playbor’” (p. 73).

There has not yet been an academic study we could locate that focuses on the economic impacts of eSports, whether on players, game companies, local and global tourism, and so on, so we do not yet have good data to answer the question of what the exchange value for playing eSports is. But it’s very clear that an important driver of eSports revenue is, simply, its viewers. As T. L. Taylor (2012), N. Taylor (2016), Jin (2010), and others (see Chee, 2006; Seo & Jung, 2016; Witkowski, 2012) point out, spectators, whether online or in person, are actively sought out by eSports players. N. Taylor (2016) goes further, and notes through a comparison of MLG events four years apart, that part of what has made eSports so successful is its “‘techniques of audiencing’ enacted through marketing strategies, data collection methods, public relations, and technological media” (p. 296). He argues that the shift to “audiencing” disrupts a dichotomous relationship between passive media consumption and participatory media (p. 304) and instead gives action and intent to both spectators and players. This significant shift ascribes participatory power to spectators (and audiences, see Smythe, 2001/2006), revealing, as N. Taylor points out, audiences are neither static nor merely constructs of media, but instead conduct affective work of their own, through fan support of teams (online or off), viewership, “likes,” and other forms of cheerleading (N. Taylor, Jenson, & de Castell, 2009).

As viewership becomes increasingly important and tied to revenue streams (Celis, 2016), and given that the eSports audience is largely male (Laurent & Abboud, 2017), there are many questions to ask about who pays to view, how often, how much they invest in their viewership, how they watch and for what purposes, and who gets paid to play? For example, in an interview with *Engineering and Technology Magazine* (Boffard, 2016), Michael O’Dell, a professional player on Team Dignitas, estimates: “…the average salary for a North American player is around $50,000. And that’s salary. Tournament winnings and sponsorship and streaming money is on top. They can earn quite a bit if they are successful” (p. 67). In that league, not surprisingly, the top grossing eSports players are all male (http://www.esportsearnings.com/players), with the top male player earning over US$2.5 million in prize money alone. The top female player by comparison earned US$170,000 (http://www.esportsearnings.com/players).

Sponsorships by companies like Red Bull, Intel, Logitech and Twitch, among others can be lucrative, supporting top players with monthly stipends reportedly upwards of six figures for the top professional gamers and/or teams. This signals that in an industry that has already grown year over year by over by over 50% per annum and is expected to generate revenues close to US$1 billion by 2019 (https://newzoo.com/insights/markets/esports/), it *is noteworthy* that professional gaming remains male dominated, and very much a “boys club” that has worked hard over the past 15 years to have pro-gaming designated a “sport,” and one that is not welcoming to women. Competitive digital gameplay is a space that is vocally homophobic, misogynistic, and racist, which attempts to excuse its exclusionary utterances as simply “trash talk” (Hamilton, 2012).

For example, a top, successful *female* professional gamer, Stephanie Harvey, a *Counter-Strike* player and currently ranked as the seventh top grossing female player (US$21,000, http://www.Esporsearnings.com/players/female-players), is often harassed and threatened with threats of rape while playing. She spoke in 2016 at the GDC along with Morgan Romine, arguing that female-only tournaments “are a necessary first step to creating a more inclusive esports culture” (Messner, 2016). Romine, who is working to find ways to support women in eSports stated: “It’s a temporary strategy, and we also don’t think it’s a one-pronged strategy—there needs to be other things happening in this space to help support a greater vision of diversity” (Messner, 2016).

There are problems here, however, that this long sought-after “vision of diversity” disguises. The increased visibility of women as players and content producers conceals from view the deeper problems of gender inequality preserved and, arguably, intensified as the competition for spectatorship overtakes traditional gameplay incentives. It is not enough to increase the number of women in these new and potentially lucrative media industries; *rather*, *we need to interrogate the relative costs and benefits of participation to better understand how masculinity is enjoying a significant resurgence of control even as women may be increasingly visible* “*participants.*”

## Broadcasting Play: User-Generated Gameplay on Twitch and YouTube

Like eSports, the self-broadcasting of gameplay, on any platform (Twitch, YouTube, and Azubu) can be economically and socially rewarding—for some anyway. Postigo’s (2016) article, “The socio-technical architecture of digital labor: Converting play into YouTube money,” offers one of the more nuanced and in-depth accounts of this tension. His research focuses on video game commentary on YouTube over a 2-year period (2009–2011), where he conducted participant observation of 20 YouTube commentators and their communities. This article offers a rare glimpse into the costs and benefits of user created content by video game commentators. Among its highlights are (1) being a commentator requires an up-front investment, which Prostigo estimates is around US$5,000, just to make the videos; (2) production values for the video matters; (3) “any given video takes 10–15 hours to produce, if not more, taking into account time to game, commentate, produce, render and post…. This is not a hobby for those who are pressed for time or have extensive family responsibilities” (p. 343); (4) subscribers are a “managerial class” which “function as the basic currency…within the digital labor architecture” (p. 345). Prostigo’s work provides a view into YouTube video game commentator’s practices and the kinds of immaterial and other labor required to support that: upfront investment, networked affective labor promoting self and others’ work, and significant free or other time to produce the videos.

Prostigo does not tell us whether the 20 commentators he focused on were male or female, but as the top Twitch streamers (by subscription numbers) or the top YouTube video game commentators/Let’s Play video producers are, to date, primarily male, it’s a defensible assumption.^3^ That is not to say there aren’t female and other Twitch streamers and YouTube commentators, but Prostigo overlooks the intersections of gender, sexuality, ethnicity, and ability in his analyses of labor in digital environments. What is often missing in accounts like Prostigo’s is who is supporting the production of such digital and affective labor—who supports the initial investment in technology? Overlooked here are important questions about the fundamental means of production: Who makes the entrepreneurial gamer possible? Who cooks the meals? Who pays the rent? How do we better make visible these practices? How can we as researchers and theorists, without endlessly repeating ourselves, uncover the ways in which hegemonic masculinities are “in play”? That is, those masculinities that are “most highly valued, legitimated and respected in society,” even as they “disempower women and…subordinate other men” (Bain, 2009, p. 486). Finally, how can we be attentive to these new labor practices while still recalling that there are other powerful structures in place that are material and reproductive. As Jarrett (2014) insists: “It is important that feminist research, typically generated by women, into the specificity of affective and immaterial labour historically associated with women, is not lost in the novelty attributed to digital media” (p. 26).

## Concluding Thoughts: Who Games? and Who Loses?

Tokumitsu (2014) concludes her critique of the “voluntary” labor mantra to “do what you love” that is now ubiquitous in the digital media industries,

In masking the very exploitative mechanisms of labour that it fuels, DWYL is, in fact, the most perfect ideological tool of capitalism. It shunts aside the labour of others and disguises our own labour to ourselves. It hides the fact that if we acknowledge all of our work as work, we could set appropriate limits for it, demanding fair compensation and humane schedules that allow for family and leisure time. And if we did that, more of us could get around to doing what it is we really love. (n.p.)

Too often overlooked in the valorization of digitally enabled opportunities for creative work and play in participatory media spheres are its imbrications with capitalism’s well-entrenched structures of exploitation and inequality (Terranova, 2000). When aspirations are attained of earning a living by doing what you love, playing games, in this case, then that activity, that previously had only use value (entertainment and new forms of sociality), realizes exchange value as well, double dipping, so to speak, from both value spheres as players “get paid to play.” Might digital gameplay, however, as a boundary object seemingly held in common across both value spheres, actually be getting significantly reshaped as it is differently mobilized, “played out” and “redeemed” within these different value spheres? Such transformation, we argue, renders invisible hugely precarious, unequally rewarded aspirational labor on the one side and losses to the ludic on the other. Gamers are themselves gamed by capitalist modes of digital production bringing losses to both the playing and the *playfulness* of games as top-ranked/remunerated players shift their activities from play to public performance, subsuming intrinsic rewards of player successes to the instrumental rewards of player reputation, in economies both symbolic and material now fueled as much (or more) by spectatorship as by participation.

## Notes

1. We recognize that the category of “women” and indeed “men” is bifurcating and masks the many and variegated forms that sex and gender take. Here, we use the terms because they mark the largest identificatory categories that are “counted” globally, and because what remains true is that whether you are born “female” or identify as a woman, you are likely to be paid less than your “male” counterparts, are less likely to serve as CEO of a company, less likely to be prime minister or president and certainly will make less for playing the same sports—and games—as your male counterparts.

2. As large as that might seem, it does not come close to other sports industries, with European football worth US$30 billion and National Football League (NFL) football worth US$11 billion in 2016 (Deloitte, 2016). Further, even if eSports were to triple its growth rate, according to Deloitte (2016), it is still worth only 1% of the wider global sports industry.

3. It is not easy to get reliable information on YouTube stars that specifically focus on Let’s Play videos, though some of it is organized by numbers of subscribers (http://letsplay.wikia.com/wiki/List_of_Let%27s_Players; https://www.thetoptens.com/best-youtube-gaming-channels/) and others by gross income largely from subscribers (http://mediakix.com/2016/02/top-gaming-youtubers-channels/#gs.GYumgJ8). That said, of three independently verified and compiled lists cited, only one includes a woman in the top 20, iHasCupquake (http://mediakix.com/2016/02/top-gaming-youtubers-channels/#gs.GYumgJ8).
